# The dark side of apnea: altered 24-hour melatonin secretion in obstructive sleep apnea (OSAS) is disease severity dependent

**DOI:** 10.1007/s11325-024-03066-5

**Published:** 2024-05-27

**Authors:** Peter Karel, Mirella Schilperoord, Loes J. A. Reichman, Johannes G. Krabbe

**Affiliations:** 1 Department of Clinical Chemistry and Laboratory Medicine, Ziekenhuisgroep Twente, Almelo, The Netherlands; 2 Department of Clinical Chemistry and Laboratory Medicine, Unilabs Oost, Enschede, The Netherlands; 3 Department of Neurology, Ziekenhuisgroep Twente, Almelo, Nederland; 4 Department of Clinical Chemistry and Laboratory Medicine, Medisch Spectrum Twente, Enschede, The Netherlands; 5 Faculty of Science and Technology, University of Twente, Enschede, The Netherlands

**Keywords:** Salivary melatonin, OSAS, Obstructive sleep apnea, Circadian rhythm

## Abstract

**Purpose:**

Melatonin aids in the synchronization of the circadian rhythm to the external environment. Few studies have tried to elucidate the relationship between melatonin and obstructive sleep apnea syndrome (OSAS). These often include few patients, do not differentiate between OSAS severity and/or do not analyse a 24-h melatonin profile. This study set out to investigate disease severity dependent differences in 24-h salivary melatonin secretion of OSAS patients compared to a reference population in a retrospective design.

**Methods:**

24-h salivary melatonin profiles of 169 OSAS patients were analysed (55 light, 66 moderate, 48 severe) as well as 91 reference patients. Several aspects of the melatonin curve were analysed and stratified according to OSAS severity. Parameters included: dim light melatonin onset (DLMO), time of returning below DLMO (DLMOoff), peak melatonin concentration and time, and total melatonin exposure.

**Results:**

Significant effects were corrected for confounding by age and sex using linear regression. Our analysis shows that, compared to reference and in a disease dependent manner, OSAS patients have a significantly lower 24-h melatonin curve, lower melatonin peak concentration, lower total melatonin exposure and a smaller proportion of patients reach DLMO. The differences in peak melatonin production and total melatonin exposure were resistant to confounding by age and/or sex.

**Conclusion:**

This study describes clear OSAS severity dependent abnormalities in melatonin production in OSAS patients, independent of sex and/or age. Future research should indicate whether oral melatonin supplementation has beneficial effects in OSAS patients with attenuated endogenous melatonin production.

## Introduction

Melatonin, a hormone secreted by the pineal gland, is one of the key players in regulating circadian rhythm [1, 2]. Melatonin secretion is tightly controlled and primarily occurs during the night. It therefore acts as a "zeitgeber”, aiding in the synchronization of the circadian system and the environment, it plays a vital role in regulating sleep–wake patterns, promoting sleep onset, and enhancing the sleep quality [3, 4]. Furthermore, melatonin possesses antioxidant, and immunomodulatory properties, which may contribute to its protective effects against various diseases [5, 6].

Desynchronization of melatonin excretion and the external environment can lead to circadian rhythm disorders such as advanced and delayed sleep phase syndrome [7]. The diagnostic work-up of these disorders can include serial measurements of melatonin to determine the dim light melatonin onset (DLMO), the time in the evening at which melatonin rises above a certain threshold (i.e. 3–4 pg/mL in saliva or 10 pg/mL in plasma) [8]. Despite its central role in sleep and the circadian rhythm, it is surprising that not more effort has been invested in identifying which other sleeping disorders may include patient (sub)groups with altered endogenous melatonin production.

Obstructive sleep apnoea syndrome (OSAS) is a prevalent sleep disorder characterized by episodes of upper airway collapse during sleep, leading to apnoea and hypopnoea. This can result in nonrestorative sleep and excessive daytime sleepiness (EDS). Moreover, OSAS is associated with the development of significant health risks including hypertension and cardiovascular disease [9, 10].

Some studies on melatonin production in OSAS patients have been published. Reporting results such as no differences in DLMO [11, 12], shifted circadian rhythm to late morning [13] or lower peak melatonin production [13, 14]. However, these studies include few patients, do not differentiate between OSAS severity and/or do not analyse a 24-h melatonin profile, potentially missing defining characteristics of endogenous melatonin production. A thorough understanding of potential alterations in melatonin production underlying these disorders may identify patients that can benefit from suppletion and/or light therapy or even shed light on disease aetiology.

Our sleep centre is one of few in the country that routinely uses 24-h salivary melatonin profiles to aid clinical decision making. As such, we have generated a relatively large database of melatonin measurements in patients with different sleeping disorders. The current study aims to confirm differences in endogenous melatonin secretion in OSAS patients, identified in previous studies described above, and to stratify these differences according to OSAS severity. To this end, we analysed salivary melatonin production in a cohort of 169 OSAS patients using a retrospective cross-sectional design.

## Methods

### Data search

All 24-h melatonin profiles of patients presenting to department of Neurology of Ziekenhuisgroep Twente hospital (Almelo, The Netherlands) in the period of 2016–2020, were extracted from the laboratory information system (GLIMS Clinisys, Belgium). Simultaneously, sex, date of birth, and date of sampling were extracted. Profiles from patients < 18 years of age at time of sampling and incomplete profiles were excluded from further analysis. Sleep disorder diagnosis and OSAS severity were extracted from the electronic health record, Hix (Chipsoft, The Netherlands) using CTcue (IQVIA, The Netherlands). This clinical data was checked and supplemented manually by the authors where needed. Authors cross-checked a subset of diagnoses to prevent inconsistency in diagnosis interpretation between individuals. Patients were excluded when under oral melatonin supplementation during saliva sampling, patient received any non-OSAS related sleeping disorder diagnosis, and/or patient reported currently working in shift work.

### Clinical aspects

OSAS was diagnosed using poly(somno)graphy. Diagnosis and severity of disease were determined by a consulting pulmonologist and based on the apnoea-hypopnea index (AHI) and the severity of hypersomnolence during the day, according to Dutch national guidelines. In short, light OSAS: AHI = 5–15 and/or limited daytime sleepiness; moderate OSAS: AHI = 16–30 and/or moderate daytime sleepiness; severe OSAS: AHI > 30 and/or severe daytime sleepiness. In this study the conclusion of the pulmonologist was extracted from the electronic health record.

### Melatonin collection and analysis

The patients were instructed to collect samples in dim lighting, to avoid bright screens (such as televisions) up to 1 h before sample collection, to avoid physical exertion, not to brush their teeth, and to avoid several types of food. Patients received a set of 12 saliva collection tubes (Sarstedt Salivette, Germany) and instructions to collect saliva in an at home setting by chewing on the cotton roll inside for at least 1 min every 2 h starting at 5 pm. Samples were delivered/sent by mail to Medlon laboratory (Enschede, The Netherlands).

Saliva was stored at -20 °C until analysis. Melatonin concentration was determined using an in-house developed LC–MS/MS method, performance of which, compared to the LC–MS/MS test it was based on and a previously used radioimmunoassay are described elsewhere [15, 16]. In short, internal standard melatonin-d7 (CDN isotopes, Canada) was added to samples and solid-phase extraction was used to extract melatonin from saliva using Chromabond C18 f—3 ml/200 mg columns (Macherey–Nagel, Germany). Eluate was evaporated and reconstituted in 10% methanol. The reconstituted sample was separated using C8-reversed-phase chromatography (column: Zorbax SB-C8, 3 × 100 mm, 3.5 µm) followed by electrospray mass-spectrometric detection on a QTRAP-3200 (ABI Sciex, USA) using the following transitions: 233.3 > 174.2 and 233.3 > 130.1 for melatonin and 240.2 > 178.0 for melatonin-d7. The assay has a limit of quantitation of 1 pg/mL and is calibrated every run with a certified reference material (Cerilliant, USA) to ensure trueness.

### Statistics

All analyses were performed using IBM SPSS 23.0 (IBM, USA), and all Figures were prepared using Graphpad Prism 10 (Graphpad Software, USA). The following parameters were analysed: 24-h curve, percentage of patients reaching DLMO, DLMO (time when melatonin concentration > 4 pg/mL), DLMOoff (time in the morning when melatonin drops below 4 pg/mL), time spent above DLMO, peak melatonin height, peak melatonin time, area under the curve (AUC: measure of total melatonin exposure). All data was first analysed in four groups (reference, OSAS light, OSAS moderate and OSAS severe) after which visual inspection of the data warranted further analysis of just the OSAS groups. 24-h melatonin profiles were analysed using repeated measures ANOVA followed by post-hoc Tukey HSD (4 groups)/LSD (3 groups) correction for multiple testing. Percentage of patients reaching DLMO and distribution of sex across groups were analysed using Chi-Square analysis. For all other parameters univariate analysis followed by post-hoc Tukey HSD or LSD corrections, was used as above. Lastly, linear regression analysis was performed on significant parameters (peak melatonin, AUC, DLMO and time above DLMO) by stepwise addition of possible confounding variables (age/sex).

## Results

### Study population

**Fig. 1:**
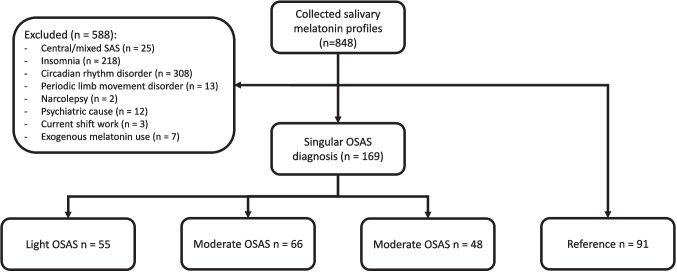
Overview of included patients

848 melatonin profiles were collected. Of these, 169 patients were diagnosed with OSAS (55 light, 66 moderate, and 48 severe). Additionally, of 91 patients no physical cause for their symptoms was found. Therefore, these were used as a reference group, as a circadian rhythm disorder and OSAS was ruled out. A detailed overview of included/excluded patients is shown in Fig. 1. Table 1 describes patient distribution across groups, average age, and percentage of male participants. Univariate analysis revealed a significant difference in age between groups (F_3,256_ = 15.92, *p* < 0.001). Post-hoc analysis demonstrated a younger reference group compared to all OSAS groups (light: mean difference (MD) = -7.97, *p* < 0.01, moderate: MD = -12.69, *p* < 0.001, severe: MD = -11.52, *p* < 0.001). When comparing only the OSAS groups, no significant age differences were observed (F_2,166_ = 2.90, *p* = NS).

**Table 1:** Subject characteristics

	Reference	OSAS (light)	OSAS (mild)	OSAS (severe)	*p* (all groups)	*p* (OSAS groups)
*n*	91	55	64	48	-	-
%Male	62	53	61	73	*p* < 0,001^#^	*p* < 0,05^#^
Average age	42	51	56	55	*p* < 0,001^$^	NS^$^

^#^: *p* values calculated using chi-square analysis, $: *p* values calculated using univariate ANOVA. For post-hoc results see text

Furthermore, we found a significant difference in the distribution of sex among all groups (χ^2^ = 15.17, *p* < 0.001) and when analysing only the OSAS patients (χ^2^ = 4.36, *p* < 0.05).

### Melatonin profile characteristics

#### 24-h melatonin profile

**Fig. 2:**
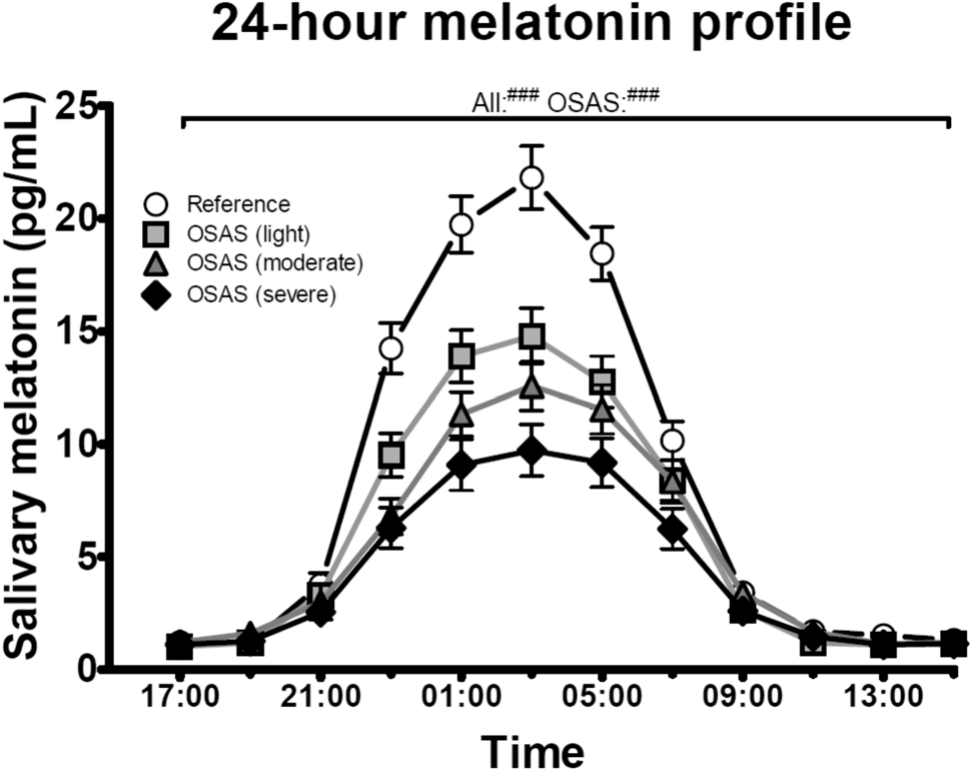
Lower 24-h salivary melatonin secretion in OSAS patients*.* Data represented as average ± SEM. Melatonin secretion profile of reference group (open circles), light OSAS (light grey squares), moderate OSAS (dark grey triangles), and severe OSAS (black diamonds). Overall repeated measures ANOVA results for all groups and only OSAS groups (^###^*p* < 0.001). For post-hoc analysis results see main text

24-h melatonin profiles of all groups are presented in Fig. 2. Repeated measures ANOVA revealed a significant diagnosis*time interaction (F_33,2850_ = 10.27, *p* < 0.001). Post-hoc analysis indicated significantly higher melatonin concentration in the reference group compared to all OSAS groups (light: MD = 2.29, *p* < 0.001, moderate: MD = 2.89, *p* < 0.001, severe: MD = 3.89, *p* < 0.001), but no differences between the different OSAS groups. However, as demonstrated in Fig. 2, there seems to be a ‘severity-response’ effect between the OSAS groups. Therefore, these groups were analysed without the reference group. In support of this observation, repeated measures ANOVA revealed a significant OSAS severity*time interaction (F_22,1815_ = 2.50, *p* < 0.001), with post-hoc significant difference between light and severe OSAS (MD = 1.60, *p* < 0.05). Interestingly, when analysing all 848 profiles, repeated measures ANOVA shows significantly lower melatonin secretion in male patients (372 males vs 476 females, F_11,9306_ = 10.22, *p* < 0.001, data not shown).

#### DLMO, DLMOoff and time above DLMO

**Fig. 3:**
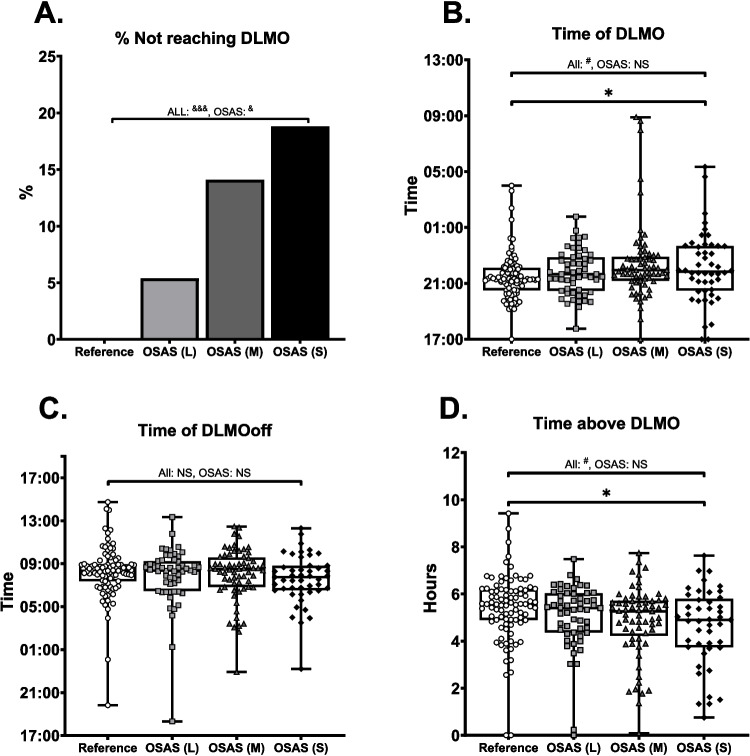
Differences in DLMO related parameters in OSAS patients*.* A: data represented as % of patients, B-D: data represented as boxplots with the box consisting of 25th, 50th (median) and 75th percentile of data, whiskers indicate minimum and maximum values. Individual patient data is plotted as open circles (reference), light grey squares (light OSAS), dark grey triangles (moderate OSAS), or black diamonds (severe OSAS). A: percentage of patients not reaching DLMO, B: DLMO time of remaining patients, C: DLMOoff time, D: total time spent above DLMO. Statistics: &: chi-square, #: univariate ANOVA, *: Tukey HSD post-hoc. Number of symbols denotes level of significance (single: *p* < 0.05, double: *p* < 0.01, triple: *p* < 0.001), NS: non-significant. L: light, M: moderate, S: severe

9.5% of all patients did not achieve DLMO. By definition, all patients included in the reference group achieved DLMO. In the OSAS groups, DLMO was not achieved by 5.4%, 14.1% and 18.8% for light, moderate and severe OSAS respectively (Fig. 3A). Chi-square analysis revealed this effect was significant when analysing all groups (χ^2^ = 15.76, *p* < 0.001) or only OSAS patients (χ^2^ = 4.17, *p* < 0.05).

Univariate analysis of DLMO of the remaining patients revealed a small but significant right-shift in OSAS patients compared to the reference population (Fig. 3B, F3,257 = 3.31, *p* < 0.05). However, post-hoc, this effect was only significant between reference and severe OSAS groups (MD = 0.49, *p* < 0.05). In line with this, when analysing only the OSAS groups, no main effect was observed (F_(2,164)_ = 3.89, *p* = NS).

Additionally, as shown in Fig. 3C, there was no difference in DLMOoff when analysing all groups (F_3,254=_3.82, *p* = NS), or only OSAS groups (F_2,164_ = 1.19, *p* = NS).

In line with the above, univariate analysis of time spent above DLMO (Fig. 3D) was slightly different when analysing all groups (F_3,252_ = 7.29, *p* < 0.05). Post-hoc analysis revealed a slightly longer time above DLMO for the reference group compared to severe OSAS group (MD = 0.78, *p* < 0.05). However, when analysing only OSAS groups, no significant effect was observed (F_2,162_ = 1.04, *p* = NS).

#### Peak melatonin and total melatonin exposure

**Fig. 4:**
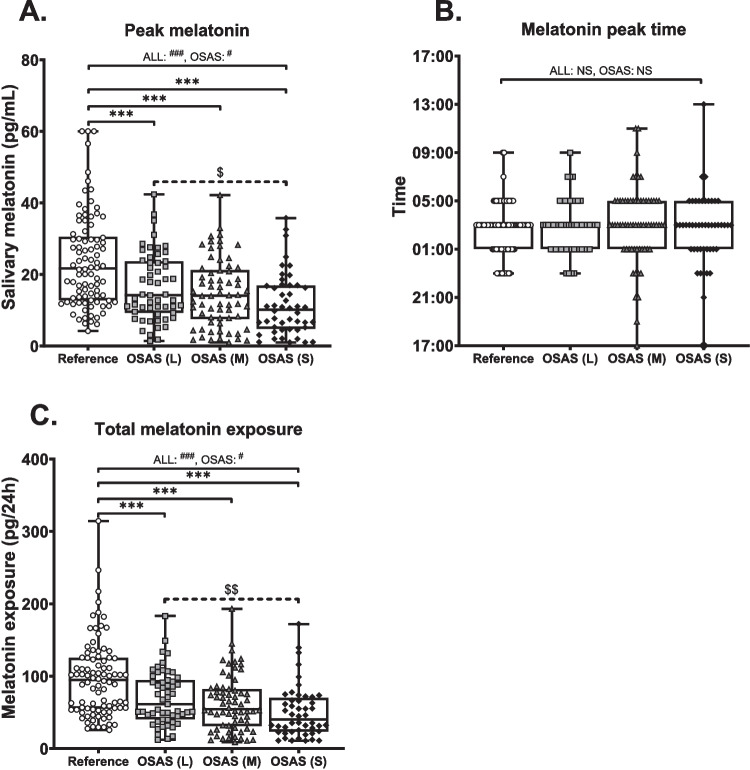
Reduced melatonin peak height and total melatonin exposure in OSAS patients*.* data represented as boxplots with the box consisting of 25th, 50th (median) and 75th percentile of data, whiskers indicate minimum and maximum values. Individual patient data is plotted as open circles (reference), light grey squares (light OSAS), dark grey triangles (moderate OSAS), or black diamonds (severe OSAS). A: peak salivary melatonin, B: time of peak melatonin, C: Total melatonin exposure (measured as area under the 24-h melatonin curve). Statistics: #: univariate ANOVA, * and solid line: Tukey HSD post-hoc, $ and dashed line: LSD post-hoc. Number of symbols denotes level of significance (single: *p* < 0.05, double: *p* < 0.01, triple: *p* < 0.001), NS: non-significant. L: light, M: moderate, S: severe

Figure 4A shows decreased peak melatonin concentration in OSAS groups compared to the reference group. Indeed, univariate analysis shows a significant main effect (F_3,256_ = 16.9, *p* < 0.001). Post-hoc analysis revealed a significantly lower melatonin peak in all OSAS groups compared to reference (light: MD = 7.29, *p* < 0.001, moderate: MD = 8.96, *p* < 0.001 and severe: MD = 12.29, *p* < 0.001), without significant differences between the OSAS groups. However, Fig. 4 suggests a ‘severity-effect’ relationship within de OSAS groups. When only comparing OSAS patients the main effect persisted (F_2,166_ = 3.86, *p* < 0.05), with a post-hoc difference between light and severe OSAS (main difference = 4.99, *p* < 0.01). In contrast, as shown in Fig. 4B, no significant differences were observed in time of melatonin peak when comparing all groups (univariate analysis: F_3,256_ = 0.61, *p* = NS) or OSAS groups only (univariate analysis: F_2,166_ = 0.66, *p* = NS).

In line with differences in peak melatonin, 24-h melatonin exposure was significantly different in univariate analysis when comparing all groups (F_3,256_ = 15.00, *p* < 0.001). post-hoc analysis revealed significant reduction of melatonin exposure in all OSAS groups compared to reference group (light: MD = 27.44, *p* < 0.01, moderate: MD = 34.65, *p* < 0.001, severe: MD = 46.55, *p* < 0.001), but no differences between the OSAS groups. However, when analysing only the OSAS groups the significant main effect persisted (F_2,166_ = 3.43, *p* < 0.05), with post-hoc analysis revealing a significant reduction in melatonin exposure between light and severe OSAS (MD = 19.21, *p* < 0.01).

### Linear regression analysis

Age is a known factor in reducing overall melatonin excretion [19]. Furthermore, when analysing our entire dataset, male sex is also associated with lower melatonin excretion. Possible confounding effects of age and sex on melatonin profiles and the observations described above were investigated with linear regression analysis using 3 models. Model 1 tested the relationship between the dependent variable and the diagnosis (reference and OSAS (light/moderate/severe) as independent variable, model 2 added age, and model 3 added age/sex as independent variables. This was done for significant parameters in univariate analysis discussed above: DLMO, time above DLMO, peak melatonin height and AUC. Data is represented in Table 2. When comparing all four groups there was no significant difference in model 1 for the time of DLMO (β = 0.15, *p* = NS). However, when modelling time above DLMO, the main effect persisted in model 1 (β = -0.31, *p* < 0.05) and no significant confounding of age and/or sex was found (Model 3: main effect: β = -0.27, *p* < 0.05, age: β = -0.11, *p* = NS, sex: β = -0.24, *p* = NS). Linear regression modelling of peak melatonin concentration showed a significant main effect in model 1 (β = -3.45, *p* < 0.001) after correcting for age and sex in model 3 this main effect persisted (β = -3.08, *p* < 0.001) even though, age was a significant confounder (β = -0.13, *p* < 0.05). However, sex did not interfere in melatonin peak height in this model (β = 1.79, *p* = NS). Similarly, AUC main effect persisted in model 1 (β = -12.19, *p* < 0.001) and stayed significant in model 3 (β = -9.48, p < 0.001) despite confounding by age (β = -0.57, *p* < 0.05) but not sex (β = 9.48, p = NS).

**Table 2:** Linear regression analysis of significant parameters

		DLMO			Time above DLMO			Peak melatonin			Total melatonin exposure		
		β	95% CI	*p*	β	95% CI	*p*	β	95% CI	*p*	β	95% CI	*p*
All groups	Model 1	0,15	-0,03—0,32	NS	-0,31	-0,56—-0,061	< 0,05	-3,45	-4,91—-1,98	< 0,001	-12,19	-17,92—-6,47	< 0,001
	Model 2 (model 1 + age)	-	-	-	-0,29	0,—-0,04	< 0,05	-3,21	-4,76—-1,73	< 0,001^#^	-11,15	-16,88—-5,42	< 0,001^#^
	Model 3 (model 2 + sex)	-	-	-	-0,27	-0,53—-0,02	< 0,05	-3,08	-4,56—-1,59	< 0,001^#^	-10,48	-16,89—-4,71	< 0,001^#^
OSAS groups	Model 1	0,19	-0,06—0,45	NS	-0,30	-0,65—0,05	NS	-3,00	-4,97—-1,04	< 0,01	-11,14	-19,18—-3,11	< 0,01
	Model 2 (model 1 + age)	-	-	-	-	-	-	-2,82	-4,79—-0,85	< 0,01	-10,26	-18,30—-2,21	< 0,05
	Model 3 (model 2 + sex)	-	-	-	-	-	-	-2,73	-4,73—-0,36	< 0,01	-9,69	-17–82—-1,56	< 0,05

Data shown are linear coefficient (β) and its 95% confidence interval (CI), and *p*-value*.* Model 1: main effect of OSAS on outcome, Model 2: main effect + age, model 3: main effect + age + sex. Analysis performed using all groups (top) and only OSAS groups (bottom). #: significant confounding by age (data in main text)

When performing the same analysis on the three OSAS groups, DLMO and time above DLMO do not show significant main effects in model 1 (DLMO: β = 0.19, *p* = NS, time above DLMO: β = -0.30, *p* = NS) in line with the univariate analyses described above. However, when modelling peak melatonin levels, the main effect in model 1 shows significant difference between groups (β = -3.00, *p* < 0.01). This main effect persists in model 3 (β = -2.73, *p* < 0.01) and no significant confounding of age and/or sex was found (age: β = -0.11, *p* = NS, sex: β = 0.95, *p* = NS). Similarly, the main effect for AUC in model 1 was significant (β = -11.14, *p* < 0.01) and remained significant in model 3 (β = -9.69, *p* < 0.05) with no significant confounding by age and/or sex (age: β = -0.51, *p* = NS, sex: β = 6.11, *p* = NS).

## Discussion

This study demonstrates significant changes in endogenous melatonin production in OSAS patients compared to a reference population. Moreover, we demonstrated an OSAS severity dependent relationship between maximum and total melatonin production. Additionally, differences in the proportion of patients reaching DLMO and more subtle changes in time of DLMO and time spent above DLMO were found. It is worth noting however that patients that did not reach DLMO could not be included in other DLMO related parameters. This indicates these parameters may not be the most robust to use in future research. These effects were resistant to correction for age and (male) sex, both known causes of reduced melatonin excretion [17]. Together, these findings suggest a need for more research into the relationship between melatonin and OSAS and potentially other, non-circadian rhythm sleep disorders.

While research in the past decades has concentrated on identifying biomarkers related to OSAS [18], very few studies have focused on melatonin. Additionally, the studies that have focussed on melatonin have not found concordant results. For instance, in contrast to our findings, Papaioannou and colleagues found no differences in DLMO times, despite only including moderate/severe OSAS patients [12]. Interestingly, in that study, only 9/22 OSAS patients and 9/22 healthy controls were able to reach a DLMO of 3 pg/mL. Butler and colleagues assessed melatonin secretion in a small group of OSAS patients under strict laboratory-controlled conditions. They described no difference in DLMO time or peak salivary melatonin [11]. However, in line with our results in saliva, peak serum melatonin was significantly lower in a cohort of 71 OSAS patients [13] and a study of 20 OSAS patients [14] compared to controls. However, none of the above studies related these changes to the severity of OSAS. One exception to this is the study by Reutrakul and colleagues that showed an OSAS severity dependent decrease of nocturnal 6-sulfatoxymelatonin (major melatonin metabolite) in urine of 56 diabetic patients which was associated with poorer glycaemic control [19]. Paradoxal to our results and some studies described above, higher levels of peak (plasma) melatonin in OSAS patients have also been observed [20]. Whether these differences are attributed to differences in cohort size and/or composition (age, gender, ethnicity etc.), study protocol, salivary versus plasma melatonin and/or differences in assay (immunoassay versus LC–MS/MS), remains unclear.

Currently, OSAS treatment uses an “one size fits all approach” with CPAP treatment as the central pillar [21]. However, because OSAS is a heterogeneous syndrome with differences in clinical manifestation [22] and pathophysiology [23], the view on this is slowly changing. Zinchuck and Yaggi reviewed several phenotypic sub-types of OSAS and their differing response to therapy [24]. While our study, due to its design, cannot prove a causal relationship between OSAS and melatonin, the relationship between the circadian rhythm and OSAS has been hypothesized to be bidirectional [25]. It has also been suggested that patients with OSAS have higher activation of the HPA (Hypothalamic–pituitary–adrenal) axis resulting in higher night-time cortisol concentrations, which may interact with melatonin secretion [26]. This suggests that there may be a role of melatonin supplements in OSAS treatment and that the largest benefits may be achieved in a sub-group of OSAS patients with severe deficiencies (precision medicine). Moreover, our study suggests determining complete 24-h melatonin profiles, as opposed to determining DLMO times, may aid in identifying these melatonin deficient OSAS patients.

Clinical studies investigating treatment of OSAS patients with melatonin supplements are virtually non-existent. Kryger and co-workers administered ramelteon, a selective melatonin receptor MT1/MT2 agonist, to OSAS patients and observed (non-significant) improvements in O2 saturation during sleep and reduced sleep latency. However, the primary goal of this study was to evaluate safety over efficacy, included only mild-moderate OSAS patients, and did not first characterize endogenous melatonin production [27]. This, in combination with our findings and the literature described above, underlines the need for a prospective study investigating melatonin supplementation in melatonin deficient OSAS patients.

While the strengths of this study stem from the number of included subjects, making it possible to differentiate between OSAS severity and to correct for confounding effects of age and sex, several weaknesses of the current study need to be discussed. Firstly, the selection of OSAS patients included in this study may be subject to bias. Patients were included in the study if they presented to the neurologist/somnologist, who then decided to order a salivary melatonin profile as part of routine diagnostics. This suggests OSAS patients who were not referred, or for whom no salivary melatonin profile was ordered, based on a clear suspicion of singular OSAS, were not included in the data. Secondly, in its current design it is impossible to ascribe a causative relationship between melatonin and OSAS, merely a correlation. Thirdly, although we were able to show that our results are robust, and resistant to statistical correction for age and thus age-related comorbidities, we did not take into account patient OSAS related comorbidities and medications that could affect melatonin levels. For instance, it is possible that OSAS patients, in a severity dependant manner, take more beta-blockers, medication known for lowering melatonin secretion [28, 29]. Lastly, the reference group used in this study was not a true healthy control group, as they also presented with difficulties sleeping, EDS or other symptoms. However, the 24-h melatonin profile of these patients was reviewed during the regular diagnostic process and found to be non-aberrant. Further (prospective) studies will be needed to address these issues and to determine the effect of melatonin supplements on melatonin deficient OSAS patients.

## Data Availability

The data that support the findings of this study are not openly available due to reasons of sensitivity and are available from the corresponding author upon reasonable request.
